# Extraction of pure components from overlapped signals in gas chromatography-mass spectrometry (GC-MS)

**DOI:** 10.1186/1756-0381-2-6

**Published:** 2009-10-12

**Authors:** Vladimir A Likić

**Affiliations:** 1 Bio21 Molecular Science and Biotechnology Institute, University of Melbourne, 30 Flemington Road, Parkville 3010, Australia

## Abstract

Gas chromatography-mass spectrometry (GC-MS) is a widely used analytical technique for the identification and quantification of trace chemicals in complex mixtures. When complex samples are analyzed by GC-MS it is common to observe co-elution of two or more components, resulting in an overlap of signal peaks observed in the total ion chromatogram. In such situations manual signal analysis is often the most reliable means for the extraction of pure component signals; however, a systematic manual analysis over a number of samples is both tedious and prone to error. In the past 30 years a number of computational approaches were proposed to assist in the process of the extraction of pure signals from co-eluting GC-MS components. This includes empirical methods, comparison with library spectra, eigenvalue analysis, regression and others. However, to date no approach has been recognized as best, nor accepted as standard. This situation hampers general GC-MS capabilities, and in particular has implications for the development of robust, high-throughput GC-MS analytical protocols required in metabolic profiling and biomarker discovery. Here we first discuss the nature of GC-MS data, and then review some of the approaches proposed for the extraction of pure signals from co-eluting components. We summarize and classify different approaches to this problem, and examine why so many approaches proposed in the past have failed to live up to their full promise. Finally, we give some thoughts on the future developments in this field, and suggest that the progress in general computing capabilities attained in the past two decades has opened new horizons for tackling this important problem.

## Background

Both gas chromatography and mass spectrometry are important analytical techniques in their own right. Electron ionization mass spectrometry is an approach that generates charged molecular fragments and measures their mass-to-charge (*m/z*) ratios [1]. Under standard conditions, electron ionization of small organic molecules produces complex but reproducible *m/z *patterns that can be related to the chemical structure of the parent molecule. On the other hand, gas chromatography excels at separation of components in complex mixtures, and is particularly well suited for the analysis of thermally stable compounds of low polarity [2]. The combination of gas chromatography and mass spectrometry allows for highly sensitive analysis of complex mixtures, and is routinely used in biochemical [3-6], medical [7-10], agricultural [11] and environmental [12,13] research, as well as in various industrial applications [14]. A surge of interest in GC-MS has been fueled by recent biomarker and metabolite profiling studies [6,11,15-24], and the potential of GC-MS to contribute to systems biology studies [25-27]. To this end GC-MS has been used for metabolic profiling in plants [11,15], bacteria [16,21,22], yeast [17,18] and biological fluids [19,20,23,24].

The ever increasing scope of GC-MS applications is opening new challenges in data processing and analysis [3,6,28]. GC-MS experiments on complex biological and environmental samples may result in hundreds of signals and the detection of many compounds in parallel. For example, Fiehn and co-authors have quantified 326 metabolites in *Arabidopsis thaliana *leaf tissue extracts [15]. In an independent GC-MS study of *Arabidopsis thaliana *leaves, Jonsson and co-authors detected 497 unique chemical components in five different genotypes [29]. When such complex samples are analyzed, incomplete chromatographic separations are often observed (note that this is also expected theoretically [30,31]). This manifests itself as the overlap of chromatographic peaks, which in turn makes the extraction of pure components and their mass spectra (required for unambiguous component identification) challenging. Currently, the most accurate analysis of complex GC-MS data sets can be achieved by an expert operator, however this is both time and labour intensive. The need to improve analysis times by speeding up the separation by gas chromatography without sacrificing the ability to separate/identify individual components is putting additional pressure on data processing methods.

Over the past 30 years a number of approaches for the extraction of pure components from overlapped GC-MS signals were proposed. This includes empirical methods [32-36], comparison with library spectra [37,38], differential methods [39-42], eigenvalue analysis [43-49] and regression analysis [50-54]. Some time ago methods for the extraction of pure components were reviewed [55]. The scope of GC-MS applications has increased significantly in past years, and a review of previous work seems timely. Here we first discuss the nature of GC-MS data and the problem of signal overlap which arises from co-eluting components. Subsequently, we review the most prominent approaches for the extraction of pure component signals proposed in the past, and give some thoughts on future developments in the GC-MS data processing field.

### The nature of GC-MS data

In a typical GC-MS setup, the eluate from the gas chromatographic column is led directly into the mass spectrometer ion source, and the mass spectrometer records *m/z *intensities in the repetitive scanning mode. This results in *R *mass scans recorded during the time of the experiment, at times *t*_1_, *t*_2_, ..., *t*_*R*_. Each mass scan can be converted into a series of *N m/z *intensities defined by the mass vector **m **= (*m*_1_, *m*_2_, ..., *m*_*N*_), where each *m*_*i *_corresponds to one *m/z *"channel". This results in a series of mass spectra, defined by the mass vector **m**, and taken at times *t*_1_, *t*_2_, ..., *t*_*R*_. As the mixture components elute from the chromatographic column their concentrations change, and the mass spectra of this continuously changing mixture are recorded.

Consider analysis of a mixture containing *K *pure components, whose mass spectra are *δ*_1_, *δ*_2_, ..., *δ*_*K*_:

(1)

The above equations can be written more concisely by introducing the matrix Δ,

(2)

Let **C **be the matrix of concentrations of *K *pure components over the time of the GC-MS experiment, sampled at points *t*_1_, *t*_2_, ..., *t*_*R*_. These concentrations could themselves be arranged into a two-dimensional matrix C, where each row corresponds to one sampling time point:

(3)

The assumption of the linear mixture model is that the observed mass spectrum is a linear combination of pure component mass spectra [44,45]. This is a robust assumption, implying that mass spectrum observed at each mass spectral scan is the result of a linear combination of the component mass spectra, where the weighting coefficients are given by the concentrations of individual components. Therefore, the mass spectrum observed at time *t*_*i *_is:

(4)

where *c*_*i*1_, *c*_*i*2_, ⋯, *c*_*iK *_are the concentrations of *K *pure components at time *t*_*i*_, and *δ*_*k *_refers to the mass spectrum of the pure component *k*, given in the equation (1). The equation (4) can be rewritten more succinctly in the matrix notation,

(5)

where the matrices C and Δ are given by the equations (3) and (2), respectively. The matrix **S **represents the net result of a GC-MS experiment, after the transformation of raw data scans into *m/z *intensities over channels defined by **m**:

(6)

In the above matrix, each row represents the mass spectrum  recorded at time *t*_*i*_. In the mathematical notation,

(7)

for *i *= 1, 2, ..., *R*. The total of *R *mass spectra of the eluting mixture are recorded at times *t*_*i*_. Equation (4) shows the same quantity, , written in a more explicit form.

In summary, the matrix given by the equation (6) represents the net result of a GC-MS experiment. Conceptually, this matrix can be viewed as the product of two matrices, the matrix **C **(equation (3)), whose columns contain concentrations of pure components as a function of elution time, and the matrix Δ (equation (2)), whose rows contain mass spectra of pure components. For the sake of simplicity, experimental noise was neglected in the above considerations.

### Total ion chromatogram (TIC)

A single column of the complete GC-MS data matrix, equation (6), is called *ion chromatogram*. It represents the elution profile of a single *m/z *channel. In practice, the GC-MS data matrix is usually viewed as the sum of its ion chromatograms, which is called a total ion chromatogram (TIC). A TIC is obtained by summing the complete GC-MS data matrix (6) along its columns,

(8)

where  for *i *= 1, 2, ..., *R*. A comparison with the equation (7) shows that *a*_*i *_is the sum of intensities present in the mass spectrum  (or equivalently, the mass spectral scan taken at *t*_*i*_), summed over all measured *m/z *values.

### The problem of signal overlap

Dynamic interactions of solute with mobile and stationary phases, as well as solute axial diffusion, lead to broadening of component zones as the solute progresses along the column [2,56]. These kinetic processes give rise to familiar chromatographic peaks, which represent component concentration in the mobile phase observed at the end of the column as a function of elution time. The chromatographic peaks have a complex shape, and in practice are most often modelled with the exponentially modified Gaussian function [57]. For the sake of simplicity, in the example below we assume simple Gaussian peaks. In this case, each column of the matrix **C **given by the equation (2) will contain a single Gaussian peak centered at the elution time characteristic of that particular solute component.

**Figure 1:**
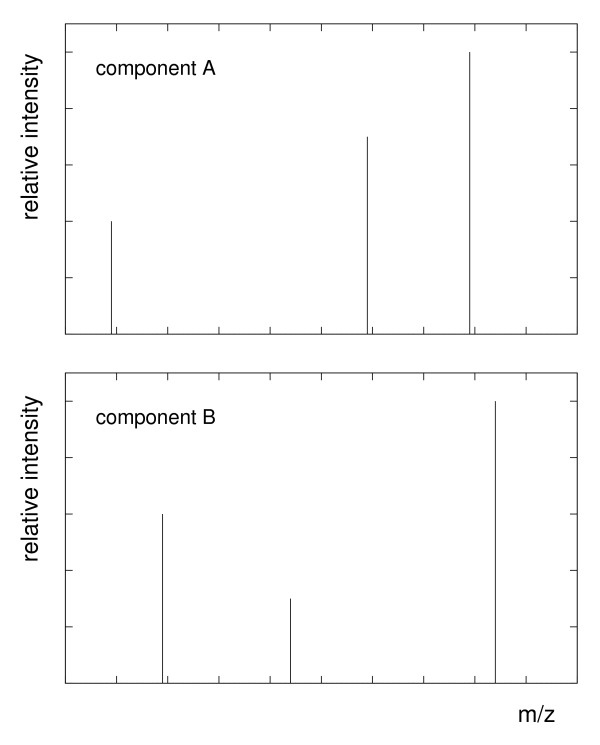
**The assumed mass spectra of pure components A and B**. The simulated GC-MS profile is shown in Figure 2.

**Figure 2:**
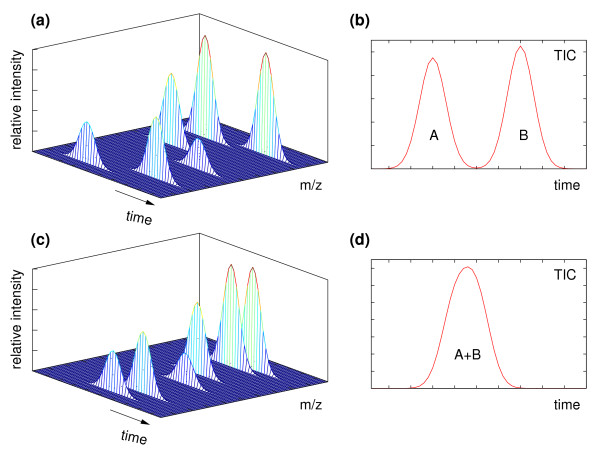
**Two scenarios illustrating the problem of peak overlap in GC-MS data**. Components A and B, whose mass spectra are given in Figure 1 are assumed to be present in the mixture. If the retention times of the two components differ significantly the observed signal will consist of two well resolved peaks, as shown in the panel (a); the panel (b) shows the corresponding total ion chromatogram (TIC). If the two components elute closely together (panel (c)), the TIC may exhibit only a single, composite peak, as shown in panel (d).

Consider a hypothetical mixture of two components A and B (*K *= 2), whose pure mass spectra are shown in Figure 1. We assume that the component A elutes from the gas chromatography column earlier than the component B (*t*^*A *^<*t*^*B*^, where *t*^*A *^and *t*^*B *^are the retention times of the components A and B, respectively). If the two components elute at significantly different retention times, they will be well resolved (Figure 2, panel (a)), resulting in two visible signal peaks in the TIC, as shown in Figure 2, panel (b). The pure mass spectra of the two components are given by the mass spectral scans taken at the apex of each component peak, and correspond to the mass spectra given in Figure 1. However, if the two components elute close in time, as depicted in Figure 2, panel (c), a severe overlap of component signals will occur. In this case a single chromatographic peak may be observed in the TIC, as shown in Figure 2, panel (d). The mass spectrum at the apex of the composite peak will be a mixture of the pure mass spectra of the two components, equation (4).

The problem of extraction of pure component signals in incomplete chromatographic separation is often called "peak deconvolution" [35,58]. This terminology is unfortunate because the term "deconvolution" denotes the inversion of a convolution process, a particular kind of integral transform encountered in the field of signal processing [59]. Extraction of pure components from overlapped GC-MS signals is both mathematically and conceptually different. However the term "peak deconvolution" has taken such deep roots in the GC-MS practice that is likely to remain a part of the GC-MS specialist's vocabulary for the foreseeable future.

A complete solution to the problem of pure components is provided by the matrices **C **and Δ, given by the equation (5). However, in GC-MS experiments only the matrix **S **is measured. It is a non-trivial problem to decompose the matrix **S **into matrices **C **and Δ; in the most general case such matrix decomposition does not have a unique solution. In practice the most important objective is often to identify retention times and mass spectra of individual components that contribute to the composite signal. From this viewpoint and under certain conditions one can sidestep the equation (5), and focus on some empirical way to resolve retention times and mass spectra of pure components. This results in two different approaches to the problem of extraction of pure signals from co-eluting components. "Empirical methods" sidestep the mathematics of the equation (5), and focus on some empirical way to resolve retention times and mass spectra of pure components, while "matrix methods" aim to find the solution of the matrix equation (5). The empirical methods typically apply the logic of a human analyst, and utilize the capacity of computers to process large amounts of data and execute repetitive tasks [32-36]. On the other hand, matrix methods aspire to a comprehensive solution of the equation (5) relying on some suitable assumptions, and usually attempt to use most if not all data points. These methods include for example eigenvalue analysis [43-49], regression [50-52] and differential analysis [39-42]. In the next sections we summarize the most prominent empirical and matrix methods proposed in the past.

### Methods for the extraction of pure components from overlapped GC-MS signals

#### Empirical methods

The method of Biller and Biemann [32] was the first widely used method for peak deconvolution. This method examines *m/z *intensities which maximize at any given chromatographic time point, or at adjacent mass spectral scans. If intensities of several *m/z *channels exhibit a maximum at the same time point, a chromatographic peak is recorded containing these *m/z *channels. This procedure results in "reconstructed" mass spectra of pure components, and is effective when two signals do not have common mass to charge ratios *and *maximize at two or more scans apart.

Colby extended the idea of Biller and Biemann by introducing more accurate estimates of peak positions, followed by binning [35]. In this approach peaks were identified as local maxima in ion chromatograms, and peak centroids are calculated from the three point quadratic fit centered at the local maximum. From this a "deconvoluted TIC" was calculated by binning the centroid intensities, in ten bins per scan [35]. The mass spectra of pure compounds were estimated by collecting peak centroids within the boundaries of the deconvoluted TIC peak. The author suggested that this method is capable of separating components which differ for only one quarter of a scan along the retention time axis [35]. In the original work, Colby demonstrated deconvolution of a single peak consisting of six components, all of which were resolved by the application of the proposed method [35].

Dromey and co-workers proposed an approach that relies on statistical analysis [33]. This method focused on finding well resolved peaks in individual ion chromatograms, ie. peaks that showed unique *m/z *relative to its neighbors. This is based on the assumption that even for heavily overlapped signals there will be some *m/z *that are unique to either of the two components. So called "singlet fragmentograms" provide information about the shape of component peaks, and this can be used to separate component signals in overlapped ion chromatograms, even for mass-to-charge ratios that occur in both overlapped components [33]. Dromey and co-authors proposed that two histograms are calculated for singlet peak positions, one recording signal maxima and the other recording total ion intensity above the noise level at these positions. The exact positions of components were determined by a parabolic least squares fit over the top five points in the sampled peak data. After this, the resolved spectrum of each component was obtained by the least squares fit to the model peak. The authors demonstrated that the proposed approach was able to detect indole acetic acid 3-methyl ester in complex GC-MS data acquired on human urine samples [33]. While this specific component did not give a visible signal in the TIC due to heavy overlap, the authors were able to reconstruct its pure mass spectrum [33].

Hargrove and co-authors reported that the method of Dromey failed to recognize weak but readily visible signals [34]. The problem was traced to the way the method calculated "peak sharpness", the property used to distinguish true singlet peaks from doublet or background signal [33]. Hargrove and co-authors proposed a different function for peak sharpness, and reported a marked improvement in the performance of the Dromey method [34].

Based on the ideas of Dromey et al. [33], Stein proposed an approach with refinements to improve the ability of the method to discern weak signals [36]. In this method the first step is the detection of individual components ("component perception"). For each "perceived" component the precise peak apex is calculated from the three point parabola fit centered on the maximum. Once the number and positions of components are determined, the mass spectrum for each component is obtained by the least-squares method similar to that of Dromey et al. [33]. An important aspect of this method is the analysis of the signal and noise features, used subsequently to aid in discerning the true signal from noise. An elaborate, empirical procedure involving analysis of all ion chromatograms is used to estimate a data noise factor [36]. This method also explicitly interpolates zero values which are found in the signal when measured intensities fall under the threshold, normally established during instrument tuning [36]. Stein has developed a PC program AMDIS which implements the proposed method [36].

#### Eigenvalue analysis

The first methods for GC-MS peak deconvolution based on the eigenvalue analysis were proposed not long after the Biller-Biemann method. In the method of Davis and co-authors, the principal component analysis was used to obtain the number of pure components in a composite signal, but not their mass spectra [43]. This approach was subsequently extended by several groups [44-48]. Ritter and co-authors proposed the eigenvalue analysis of the covariance matrix to obtain the number of pure components [44]. Knorr and Futrell proposed the method for the determination of both the number of pure components and their mass spectra based on the factor analysis [45]. A similar method was proposed by Abdallah and co-authors, who calculated "ranges" for the pure component mass spectra [46]. Roach and Guilhaus reported enhanced factor analysis which exploited the ordered nature of GC-MS elution profiles [48], based on the ideas by Meader (dubbed evolving factor analysis, EFA) [47]. More recently, variants of the eigenvalue analysis were applied to the analysis of complex plant extracts [49].

#### Differential methods

Ghosh and Anderegg proposed differential processing of GC-MS data in which *m/z *intensities for each two successive scans are subtracted [39,40]. This procedure resulted in two new data sets created from the original GC-MS spectral matrix, one with the positive and one with the negative differences in intensities. Ghosh and Anderegg reported that such differential processing resulted in pure component mass spectra, which can be used for reliable comparison with mass spectral libraries [39]. Pool and co-authors extended this work in two directions [41,42]. First, they proposed that two data sets resulting from the subtraction are combined into a single data set that resembles the original data; second, they proposed that this procedure is applied recursively until convergence is achieved ("backfolding") [41]. The authors reported that backfolding is capable of extracting pure mass spectra when signals are severely overlapped [42].

#### Library search

The first computer approaches to aid in identification of compounds in complex mixtures relied on comparing mass spectra to precompiled libraries [37,38]. This is of course limited by the scope of the available library. Moreover, when the signals overlap the observed mass spectrum will be a mixture, and the library search may fail to match any of the components from the mixture.

Gan and Liang proposed the method for the search of component mass spectra based on the observed composite signal [60]. This method first identifies potential candidates for component mass spectra, and then uses non-negative least-squares regression to calculate contributions of the assumed components to the observed, composite mass spectrum [60]. This process results in pure signals, and therefore could be viewed as a method for the extraction of pure components from overlapped signals.

#### Regression methods

Blaisdell and Sweeley proposed a procedure for the extraction of pure components based on the singular value decomposition and least squares fitting [50]. This method depends on the determination of background noise for each mass, which was assumed to be constant over 10-12 scans. Knorr and co-authors proposed a regression procedure where the full matrix representation of data, equation (6), is modelled as a function of component retention times. The least squares fit is performed to minimize the difference between the predicted and the observed data matrix, where individual ion chromatograms (i.e. columns of the matrix C, equation (3)) are modelled as Gaussian functions modified with an exponential decay function [51]. This requires that the number of components is known. The authors proposed a heuristic procedure based on the relationship between the number of components in the model and the observed changes in goodness-of-fit to determine the optimal number of components [51].

Karjalainen proposed alternating regression for the extraction of pure components from GC-MS data [52]. In this approach, **C **and Δ are initially set to random values, and the equation (5) is solved for both **C **and Δ iteratively, by applying constrains such as non-negativity and unimodal shape, until the convergence is achieved [52]. This method requires the number of components to be known, and the author proposed this to be found by trial-and-error [52]. Since multiple solutions may be obtained by convergence from random values, the repetition of the calculation from different initial values was proposed to establish the stability of the solution [52].

An iterative optimization method for peak deconvolution was proposed for the special case when one signal is embedded within another [53]. In this method, least squares are used to obtain mass spectra of pure components [53]. Shao and co-authors reported the application of the artificial immune algorithm for the extraction of pure components in GC-MS data [61] (immune algorithms are inspired by the defense processes of the biological immune system [62]). These authors used independent component analysis [63] to extract the mass spectra of pure components, and then chromatographic profiles corresponding to these pure components were extracted with an adaptive immune algorithm [61]. The method was demonstrated on simulated data, and on experimental data obtained on the pyrolysates of phenylalanine [61].

Stokkum and co-authors proposed the regression method based on a parametrized model of the data, where elution profiles are described with exponentially modified Gaussian functions [54]. In this method the data is separated into time windows, so that each time window contains only a small number of pure components, estimated from the principal component analysis [54]. In their model each component is described with three parameters determined by the nonnegative least squares fit, where the difference between the model at the parameter values and the data is minimized [54].

## Discussion

Automated extraction of pure components from co-eluting components in GC-MS data is a challenging problem. To make the problem tractable, most methods rely on implicit or explicit assumptions about the characteristics of the signal and the noise. For example, Knorr et al. [51] modelled signal peaks as exponentially modified Gaussian functions; Stein assumed that a single noise parameter derived from multiple ion chromatograms can adequately describe random fluctuations in data [36]; Colby assumed that a fixed number of bins is optimal to bin centroid intensities [35], and so on. The degree of validity of such assumptions will depend on the data at hand, and when the assumptions are no longer valid the method is likely to fail.

In addition, experimental GC-MS data may contain a range of irregularities and imperfections, confounding the problem further. For example, in a typical GC-MS experimental setup only intensities above a threshold are stored [36]. This may result in zero intensities, or entire blocks of zero intensities embedded in the data, which in turn complicates the analysis of noise. There are at least five experimental factors that collectively, and often confoundingly, influence the characteristics of GC-MS data:

1. *The nature of sample components*. More complex samples produce more signals per standard chromatographic separation run, and this results in increased peak crowding and overlap. The more severe the peak overlap the more difficult is the extraction of pure components, and this is especially the case if multi-component overlap occurs.

2. *The sample matrix*. The sample matrix can profoundly influence both the characteristics and quality of the GC-MS data. Samples of biological material can have large amounts of background chemicals which interfere with the detection of trace compounds, both through impeding the efficacy in separation/detection, and also by producing noise-like effects. Specifically, samples of urine, saliva and serum are associated with difficult sample matrices.

3. *Condition of the instrument*. Less than optimal instrument condition may result in chemical noise that is difficult to model (see below). For example, a worn out liner, a component of the GC inlet system, may deform peak shapes and affect peak resolution; a sub-optimal connection of the column may result in oxygen diffusion into the system increasing the background noise; septum bleed may result in wide humps that distort the signal baseline, and so on. In addition, mechanical problems associated with gas chromatography, such as uneven flow of the carrier gas or column packaging may have similar effects.

4. *Instrument tuning and experiment runtime parameters*. The parameters set by the operator, if not optimal, may adversely affect the quality of GC-MS data. For example, faster oven ramp rates result in shorter experiment times, but also increase peak crowding and consequently peak overlap.

5. *Instrument type*. Data acquired on different GC-MS instruments may have different characteristics (retention time resolution, m/z resolution, noise characteristics). For example, time-of-flight (TOF) instruments allow faster scan rates compared to quadrupole instruments, and typically result in higher resolution data.

Purely from the data viewpoint, the main challenges in automated signal detection include *a priori *unknown shapes of signal peaks and reliable separation of the true signal from noise. In most practical situations, the latter problem is more challenging; the question of peak shapes is amenable to empirical solutions. A number of empirical functions were successfully used for the modelling of chromatographic peak shapes in the past [57].

In GC-MS experiments a combination of true noise and chemical noise is typically observed. True noise refers to random fluctuations that originate from the limitations in instrument electronics (this type of noise is always present in instruments that use ion multipliers). On the other hand, chemical noise arises from extraneous chemical components introduced in the system unintentionally. Such components may be introduced during the sample preparation process (for example, as a consequence of derivatization), or may originate from the instrument condition (due to column bleed, for example). Therefore chemical noise is not noise at all, but unwanted signal that originates from chemical components introduced as a part of the experimental process [64].

**Figure 3:**
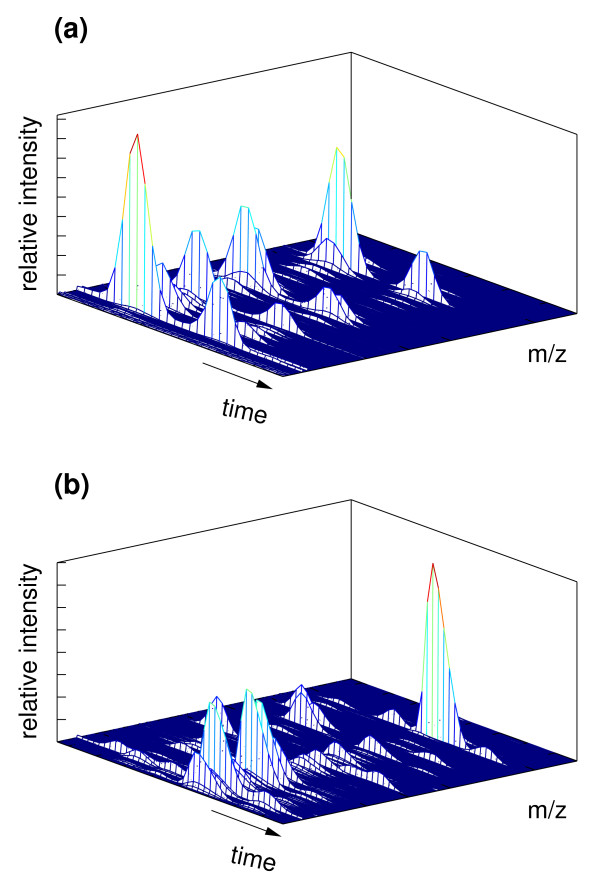
**Two fragments of experimental GC-MS data matrices, equation (6), showing signals from closely co-eluting components**. The signal peaks in the panel (a) exhibit symmetric peak shapes, while the signal peaks in the the panel (b) show slightly asymmetric peaks. This effect (dubbed "peak tailing") can originate from several instrument conditions, for example column degradation, or contaminants left in the injection port. Both data sets show a continuum between noise and weak signals, a situation typically encountered in practice.

Although the origin of noise in GC-MS experiments is well understood, in any specific experiment noise is difficult to model or account for accurately. In practice, noise may manifest itself in any number of ways. For example, the signal from chemical noise may overlap or obscure the signal of interest; alternatively the net effect may be the degradation of the signal quality due to increased background, lower signal-to-noise ratio, skewed peak shapes or distorted signal baseline. Furthermore, very low concentration components present in the sample may result in true signals that are at the level of noise. As a result, in experimental data often there is no clear separation between the signal and the noise components (Figure 3).

A review of the literature suggests that the most widely used, publicly described method for peak deconvolution is AMDIS [36] (this view is corroborated by others [65]). We speculate that this is for two reasons. First, AMDIS is probably the only method implemented in a freely available software package targeting the PC computing environment most analysts are familiar with (although it is not open source) [36]. Second, in AMDIS component detection is integrated with library matching [36], which is useful in practice.

The main weakness of empirical methods, including AMDIS, is the use of arbitrary rules and empirical parameters. For example, AMDIS divides each ion chromatogram into segments of 13 scans for noise analysis; zero abundance values are replaced based on a complicated set of empirical rules that involve several arbitrarily chosen parameters; pre-set maximum number of scans in component detection is 12; "peak sharpness" is defined by an empirical formula, which in turn features a single "noise" parameter calculated empirically, and this parameter is assumed to faithfully represent the noise; the multiplier for maximum range in peak sharpness calculation is 50; the components that do not have the sharpness within 75% of the maximum value are discarded; and so on [36]. Why exactly these numbers are chosen is impossible to justify in a truly objective way. Furthermore, the sheer number of empirical rules and parameters suggest that a systematic optimization of an empirical method such is AMDIS is difficult, and understanding fully how the parameters affect the final result is probably not a realistic goal. AMDIS was originally optimized for a specific GC-MS application [36], and subsequently applied to other systems [66,67]. However, a recent study reported that AMDIS generated as much as 70-80% false components (false positives) [58].

On the other hand, the matrix methods remain marginally used in practice, in spite of the considerable enthusiasm that surrounded many initial demonstrations. There are several reasons for this. First, most matrix methods proposed in the past were proof-of-concept demonstrations, and had failed to establish unambiguously their usefulness in real experimental scenarios. Second, often there is no intuitive picture associated with matrix methods. For example, the eigenvalue methods result in matrix decompositions of the original GC-MS data that have no physical meaning [48]. This is certainly a downside for most GC-MS practitioners, at least before the method's advantages in real experimental scenarios are clear. Finally, and related to the first point, software implementations that would allow matrix methods to be tested by a wider community and under realistic experimental scenarios are lacking. To our knowledge none of the matrix methods reviewed here were accompanied by an accessible and widely available software implementation.

Another problem is the method demonstrations in limited experimental scenarios. The first attempts to use the eigenvalue analysis for the separation of overlapped GC-MS signals were on simple binary mixtures with a limited range of *m/z *values [43-45]. Ritter and co-authors used four sets of binary mixtures (cyclohexane/cyclohexene, hexane/cyclohexane, heptane/octane, and unknown xylenes), and only 20 *m/z *values [44]. Subsequent work used more realistic but still limited experimental scenarios compared to modern standards. For example, Abdallah and co-authors used binary mixtures with 135 *m/z *values [46], while Roach and Guilhaus used a mixture of seven organochlorine compounds with a similar *m/z *range [48].

The method based on differential processing of GC-MS data was originally proposed by Ghosh and Anderegg [39,40], and subsequently developed further by Pool and co-authors [41,42]. Interestingly, the authors compared differential processing with the empirical method of Colby [35], and the regression method of Karjalainen [52], and reported that backfolding outperformed both methods [42]. Unfortunately this conclusion was based on the analysis of only a small fragment of an experimental data set [42].

The first applications of regression to GC-MS peak deconvolution were proposed not long after the first eigenvalue methods were tested [50,51]. The method of Blaisdell and Sweeley relied on both the eigenvalue analysis and linear least squares, although the original description lacked the full mathematical detail [50]. The regression method of Knorr and co-authors amounts to a mathematical decomposition of the data matrix, equation (5), where the individual ion chromatograms are modelled explicitly with modified Gaussian function [51]. This idea is clearly a viable approach for resolving multi-component overlapping signals. However, its demonstration in the original formulation was on highly simplified data compared to today's standards: binary and ternary mixtures with 30 mass spectrometry scans involving a small number of *m/z *channels [51].

The alternative regression method of Karjalainen appears to be both advanced and model-free [52]. In the original publication, the author reported two problems: with the convergence and with determining the number of components [52]. Recently, Jonsson and co-authors proposed an approach based on the method of Karjalainen [68]. In this approach each data set is divided into suitable time windows, and within each time window the overlapped signals are resolved with the alternating regression method originally proposed by Karjalainen [52]; a multivariate analysis is used to identify time windows which contain significant differences between samples [29,68]. Jonsson and co-authors also proposed an an improved method for choosing initial values that provided better convergence compared to random values, as originally proposed by Karjalainen [52].

The regression method of Gong and co-authors [53] was applied on complex plant samples; however the focus of this method was on resolving a specific type of signal overlap. An interesting outcome of this study was that signal clusters originating from co-eluting components should be analyzed differently, depending on the specific nature of the signal overlap [53]. The library search method of Gan and Liang aimed to tackle both deconvolution and spectral matching simultaneously [60]. However, even in an ideal scenario, this method has strong limitations, since any component that does not have a mass spectrum in the library cannot be identified as a part of the mixture.

A method for peak deconvolution based on artificial immune algorithm [62] was reported by Shao and co-authors [61]. Their test cases involved GC-MS data obtained from pyrolysates of phenylalanine [61]; however the analysis focused on a narrow retention time range of 0.5 minutes which contained three overlapped components. The authors also compared the performance of the proposed method with the multivariate curve resolution method SIMPLISMA [69]. To our knowledge, beyond this work SIMPLISMA was not applied to GC-MS data, although it was used for resolution of co-eluting components in liquid chromatography-mass spectrometry (LC-MS) [70]. It is interesting that SIMPLISMA [69] was originally inspired by the factor analysis work of Knorr and Futrell [45].

Recently, a novel regression method was reported by Stokkum and co-authors [54]. This method borrows several strategies from the work of Jonsson et al. [29,68], including dividing the data into time windows. Applications on real and simulated GC-MS data sets under difficult co-eluting scenarios demonstrated that this method is competitive with multivariate curve resolution [29,68] at simultaneous analysis of multiple GC-MS data sets.

## Conclusion

In this work, published methods for the extraction of pure components in GC-MS data with co-eluting components were reviewed. This provides several important insights. First, in reports presenting new peak deconvolution methods, the use of realistic experimental scenarios is important. Second, for any new method, the availability of software implementation that would allow the method to be tested by a wider GC-MS community, is critical.

Perhaps a more subtle point is that most matrix methods require the number of components to be known prior to the separation of overlapped signals. This is evident in both early studies [43-46,48,51,52] as well as in more recent works [29,53,54,68], suggesting that a separate analysis of this problem is warranted. We also note that the method of Jonsson and co-authors [29,68] may provide the recipe for a systematic deconvolution of the entire data set by applying divide-and-conquer strategy, coupled with the alternating regression originally proposed by Karjalainen [52].

Although the empirical methods for peak deconvolution are currently most widely used in practice, it seems inevitable that matrix methods will dominate the future. This is evident from the application of matrix methods to the analysis of complex plant samples [53], development of new matrix-like approaches [54,61] and methods aimed to identify differences in high-throughput GC-MS data [29,68,71].

Remarkable progress in the field of general computing in the past two decades has opened new avenues for tackling the problem of peak deconvolution, and GC-MS data processing in general. Modern computer hardware is thousands of times more capable compared to the elite computing machines of twenty years ago. Several important works reviewed here were performed on (today completely obsolete) PDP-11 computers [33,50,51]. For example, Blaisdell and co-authors reported that a mere 20,000 16-bit words of core memory was available for their programs [50]. Furthermore, modern computing clusters based on commodity hardware allow even further scaling in the CPU power. The changes in the software landscape are equally drastic. For example, in their application of principal component analysis Davis and co-authors wrote their own functions for eigenvalue decomposition in the programming language BASIC [43]. Today, software platforms such as MATLAB [72], GNU Octave [73], and R [74] provide integrated environments with thousands of highly optimized mathematical and statistical functions readily available (and in the case of open source packages such as GNU Octave and R, at no cost). Moreover, a range of open source projects such as Python [75], Perl [76], and Java [77] provide general purpose programming languages with rich and well tested libraries. These developments suggest that a new era of collaborative computing, based on open standards and open source software, is about to emerge in GC-MS data processing. A similar transformation is already evident from the initiatives to standardize representations of mass spectrometry data [78], and open source packages for LC-MS data processing recently published [79,80].

## Competing interests

The author declares that they have no competing interests.
